# Social difference and relational coaching: finding new freedoms in working with identity

**DOI:** 10.3389/fpsyg.2024.1379659

**Published:** 2024-12-09

**Authors:** Tammy Tawadros, Erik de Haan, David Birch

**Affiliations:** Ashridge Centre for Coaching, Hult International Business School, Berkhamsted, United Kingdom

**Keywords:** coaching, identity, social difference, working alliance, implicated subject, compassion

## Abstract

In this article we explore some of the processes involved in dealing with Social Difference (SD) in coaching. Using examples from our own practice, we consider several factors, including the identity work involved in navigating the experience of SD in one-to-one coaching. Dealing with experiences of difference, including social class, gender, race, ability, and sexuality can invoke complicated and powerful feelings. Feelings of guilt, shame, anger, and isolation may cause impasses and ruptures in the coaching relationship. The article also sets out to test two hypotheses. Namely that working with questions of SD can enrich coaching if the Working Alliance (WA) is experienced as positive, and that issues of SD are better managed if they are discussed explicitly. We draw on social constructionist conceptions of identity, together with Rothberg’s work on the implicated subject, to facilitate inquiry into SD and identity for ourselves as coaches, as well as for our coachees. This is the work of re-examining ourselves, re-thinking who we are, and moving beyond the unwanted aspects of the self which may provoke existential feelings of guilt, shame, anger, and isolation, as accumulated through or aggravated by the current and historical woundings of oppression and social injustice. Our research tells us that it is by facing into the complicated emotions engendered by SD, that we can begin to embrace what we psychically and socially “disown.” We suggest that by recognizing our implicated selves, we may become better equipped psychologically, to be more sensitive and responsive to the impact that SD has on individuals, groups, and organizations. We discuss the ways in which a relational stance in coaching can help to provide a secure holding environment for critical and reflexive inquiry into SD, identity and the selves we enact. We conclude that a relational and implicated approach in coaching provides a wider perspective and extends the critical capacity of leaders to look within themselves and into their challenges with more congruence and ethical maturity.

## 1 Introduction

Executive or workplace coaching helps leaders to formulate and realize their goals through a series of conversations with a contracted coach (De Haan and Burger, 2014). Working in the dialogic paradigm of coaching invites self-reflection as well as shared reflection, which can, in turn, enable powerful shifts in perspective (Stelter, 2019). There is compelling evidence that such reflective conversations can be highly effective in delivering on the outcomes that are agreed, across a wide range of settings (De Haan and Nilsson, 2022). We also know that coaching conversations can be challenging and that in some cases, contracts may not be fulfilled, for example, when clients do not attend their scheduled sessions, or where they feel unsupported (Hall et al., 1999; Berglas, 2002; Ellam-Dyson and Palmer, 2022). Essential to effective coaching seems to be the *Working Alliance* (Graßmann et al., 2020), where coach and client have a strong bond and good agreement on the objectives (“goals”) and ways of working (“tasks”) in coaching. The bond, a component of the Working Alliance (WA), reflects the degree to which the coachee feels safety, affinity and warmth in the collaboration (Bordin, 1979). Forming and maintaining a strong bond is inevitably influenced by the degree to which the coach understands, and can empathize with, the coachee’s lived experience, and vice versa. In other words, the extent to which the coach (and indeed the coachee), is able to embrace the other’s different lived experience and consequent perceptions and perspectives, can be a pivotal factor in shaping the bond between coach and coachee. This article aims to shed light on a significant challenge to the WA in coaching, which limits coaching effectiveness: distancing because of Social Difference (SD) within the coaching relationship.

We use the term SD to refer to differences in race, class, ability, gender, sexual orientation, and other aspects of identity, which confer certain systemic and structural advantages and disadvantages. Both identity and social difference can be seen as constructs which are more or less stable, carrying meaning and significance that derives from societal norms and practices. Shaped by historical and contemporary power relations, we recognize institutional and social practices which maintain not only power-based structures, but also, symbolic boundaries that operate to dominate, exclude, and categorize certain behaviors, people and groups as “other.” Those people and groups deemed socially different are invariably seen as “less than” those who have relatively more social privilege including, among others, in the form of cumulative material and cultural advantage, and access to networks of information and influence. We hold an assumption that there will inevitably be many points of overlap (intersections) between oppressed and privileged identities. Intersectionality allows for a “both and” position (both race and class for example), where several different aspects of identity combine to create a unique mosaic of lived experiences. This can make for some complexity, but also potentially more nuanced effects of SD, which need to be taken account of. Coping with and moving beyond the impact of SD is often a topic of coaching, but SD is also a relevant challenge in forging a coaching relationship and making it productive. In this contribution we will focus on the latter: SD within the coaching relationship. Central to our argument is that socio-political realities and historical contexts cannot be decoupled from coaching practice, and that issues of power and SD exert an important influence on what happens between coach and coachee.

Coaching holds out the promise of positive personal chemistry and rapport, undivided attention, and unconditional support, as well as new insights and strategies produced in collaboration. A positive WA engenders shared understanding and companionship, positive feelings and joy, a sense of being accepted, understood, and “seen.” This stimulates the coachee to make personal and professional changes which impact their effectiveness in the workplace. A strong WA enables repeated and deepened moments of connection, increasing responsiveness to others and the willingness to take risks. However, a less positive alliance may also emerge (Day et al., 2008), where misunderstandings or other misapprehensions may lead to a dynamic of missed connection, distancing, and lack of compassionate responsiveness, leading to ruptures and impasses. Where a coachee experiences a feeling that they may in some way be unworthy, not sufficiently understood, or somehow pressured by their coach, this may lead to distancing in their relationship, which in turn can cause the coachee to withdraw from the relationship or to pull out of the coaching contract altogether. Coaching inevitably reproduces the power differentials and social interactions that exist in society, inside the microcosm of the coaching room. To the extent that these reproductions, mirroring, or transferences can be understood and worked through, coaching holds great potential. It is through understanding, surfacing and attending to precisely these negative experiences of the WA that they can become a source of reflection and change. SD within the WA can foster richer reflections and generate more options to reach desired goals, as we have argued elsewhere (De Haan, 2019; Tawadros and Birch, 2023).

We find that where SD is concerned, the “content” and “process” of coaching can become rather entangled. Ruptures in the reflective process, and ruptures in relationship resulting from SD are hard to distinguish and can be mutually reinforcing. Any experience that involves a loss in understanding, connection, or respect, not only make us feel more alone, but may also serve as a reminder of present and past hurts around SD. This relates to the transferential aspect of relationships: the fact that every new relationship we form is at once novel and unique, as well as recalling memories and expectations of past relationships. Both coach and coachee carry experiences of previous relationships into the coaching relationship. These might be about not being able to work together on the issues, and a sense of having different lived experiences which are not being bridged in a session, emphasizing the social differences between them. SD can therefore play a major role in distancing on its own, but it also combines with other relationship dynamics, to seed or grow feelings of doubt about the value and effectiveness of the coaching endeavor, as well as about the current coaching relationship. As a result, any sense of distancing in the session can register strong and deeply felt emotion within the coachee and coach, and be experienced as grounds for feeling unwelcome, misunderstood, or unconnected.

When we experience this type of distancing in coaching, we may be reminded of those elements of our “identity” that in some way stand in the way of being able to relate to the other person or of being accepted. We may suddenly experience our SD, our identity as gay or Black or working class, and other important and salient aspects of our identity that make us socially different, in very visceral and powerful ways. This can make it difficult to stay in relationship and to carry on with our learning and discovering. We believe it is at this juncture that the coach and coachee have an opportunity to do difficult but productive work together, if they are able to bridge the distancing in their relationship and can make some of their SD explicit. Empathy and openness about SD help to convey the coach’s willingness to recognize the coachee’s distinctive identity, and to appreciate their coachee’s highly personal, lived experience. Additionally, self-awareness and self-compassion on the part of the coach, enables them not only to honor their experience and uniqueness, but also to show compassion for the coachee’s hurt and to stand in solidarity with their experiences of suffering, thereby underscoring their shared humanity and affirming a willingness to act in support of them. These are the relational conditions that work to deepen connection and trust, lessening the potential for identity and SD to become a barrier in the WA, or a defensive bulwark against contact with an array of complicated feelings and the replay of traumatic histories. Paradoxically, exploring, and staying with the themes and dynamic underplay of identity, SD, and distancing seems to open up a more productive curiosity in the coaching relationship.

### 1.1 Case example to set the scene

One of us once coached Mark, a senior director in the Health Service who told him early on in the first meeting, “I can see from your bio that you have been trained in the Freudian school. Now I know Freud was homophobic, so I am not sure if we will get on.” I remember feeling a range of emotions, including quite prominently a sense of rejection, but I also knew this was going to be a key moment in our relationship. Among those emotions I do not remember when “gratitude” emerged, but I think it was quite early on. This coachee was able to mention a SD and a possible obstacle for our work at a very early stage and was willing to listen to my response to his mention. I shall always remember the moment and the “second chance” I felt I had been given, before we went on to have eight very productive sessions. One of my responses initially was also intellectual: from reading Freud I know he was not critical of homosexuality at all; in fact, he hypothesized that we are all bisexual—and offered good evidence for this from his psycho-analysis practice. I knew I could not say all this, or any of this, as it would only be antagonistic to my client, and so I have not shared it at any time during our collaboration. However, I did have opportunities to come back to my client’s statement. In fact, I was already reminded of it when later in the same first session he mentioned his goals. The main one was that he wanted to be better recognized within and more supported by his peer group of service directors. It quickly emerged that most were men in that group and mostly heterosexual. They somehow were, or were seen by him as, more “brash” and “dominant” than my client. I thought and later mentioned as a hypothesis that he may have taken me for one of them at our very first moment of meeting. This led to fruitful conversations and an opportunity for him to share a lot more about what straight men had done to him in the past, and about how he often felt a lack of recognition out of a sense of being different and somehow treated pejoratively in the workplace.

In many societies, there is considerable concern about persisting social injustice. The global legacy of colonial and imperial geopolitics which led to the overpowering of many peoples by military and economic means, and the ongoing exploitation and oppression of marginalized and minoritized groups continues to exact a heavy toll on freedom, humanity, and our ability to relate and collaborate. Through the influence of liberation movements (Freire, 1970), the growing influence of social justice movements and of globalization, we have come to renew our commitment to the power of a common humanity. Many communities and organizations are working to overcome the impacts of social exclusion, by processing the pain and consequences of oppression. In recent decades, many organizations have come to recognize the value of diversity, its impact on the bottom line and on the potential for innovation and growth. It seems that managed well, difference and diversity can indeed be highly profitable in business (the so-called diversity dividend; Gompers and Kovvali, 2018). We are also seeing an increasing emphasis on environmental and social governance and a paradigm shift away from a model of shareholder profit, toward a broader responsibility for a wider group of stakeholders and communities. Rather than operating as inequity regimes, that produce inequality and social differentiation, many organizations are seeking a role in progressive social change. This is a shift which further underlines the importance of attending to SD in organizations and in executive coaching.

Our *first hypothesis* therefore is that SD may make the work of coaching harder but also promises a high return if the WA is experienced as positive. Support for this hypothesis can be found in Boyce et al. (2010) for demographic factors and De Haan et al. (2016) for personality factors.

Our *second hypothesis* is that SD can be better managed if it is explicitly surfaced, named and reviewed by coach and coachee, and become part of the coaching conversation. This is the idea of actively “broaching” SD (Bayne and Branco, 2018; Day-Vines et al., 2021). Writing specifically about race, McKenzie-Mavinga (2009) goes further and describes a “Black empathic” approach where the coach pays particular attention to the profound cultural influences of racism. This approach advocates not only broaching and exploring the area of SD with the coachee but also grounding these explorations in a recognition of the ways in which racism permeates the psyche and impacts many dimensions of experience. In McKenzie-Mavinga’s view, this requires the therapist, or indeed the coach—whether white or Black—to recognize their clients’ and their own defenses against the trauma associated with the operation of oppression and concomitant privilege.

In this article we hope to test these two hypotheses and develop a model for working with SD and the WA in coaching. We will argue that a *relational stance* can help with this challenging aspect of workplace coaching. For us a relational stance means an ongoing effort by the coach to help make their here-and-now relationship with their coachee as explicit and open to reflection as possible (De Haan, 2008; Cavicchia and Gilbert, 2018). In addition, we will outline some ways in which coaches might approach the exploration of identity and SD and use the notion of the implicated subject. We will suggest that such explorations can lead to identity work, that is, to changes in the way the coachee and coach perceive and enact their identities, in a way that supports the coaching endeavor.

## 2 Theoretical background

### 2.1 Competing and conflicting narratives about identity

Inevitably, the ways in which we conceive of and speak about identity can themselves become polarized, and it is possible to discern the influence and indeed a battle of ideas elaborated and expressed in these narratives and debates. A full account and examination of identity is beyond the scope of this article. However, we believe it is important to consider some of the prevailing narratives. One such narrative tends to characterize our political and social engagement *as consumers* of goods, services, rights and identities, as opposed to engaging *as citizens*, who have a stake in society, and global sustainability, with the capacity to act collectively. Exploring the potential of the notions of consumer and citizen, Alexander and Conrad (2023) are less concerned with psychological and social processes underpinning narratives of identity. However, they offer an important argument, namely that making a shift to thinking of ourselves as citizens, as opposed to subjects, consumers, or indeed citizen-consumers, holds within it the possibility of taking up responsible collective action. We return to the relationship between identity, identity work and action in the sections below.

In a consumerist world, where the idea of identity as a precious individual gift and a right is predominant, it can often seem that we have fetishized, individualized, and commodified our very definition and understanding of the self. We are continually exhorted to express, invent, and reinvent ourselves through our look, our belongings, and the consumer choices available to us. In this narrative of identity, we are primarily individual consumers capable of expressing and transforming our identity through what we consume in the way of goods, services and products, both material and intangible.

At the opposite end of the spectrum of narratives about identity, we find a conceptualization that is more essentialist, with the notion of identity as core and natural, if not fixed. In this narrative space, some elements of identity are essential and deterministic, if not immutable. Knowing the social value ascribed to our identity is seen as key to living a positive life and enjoying social equity. Taking pride in a minoritized and marginalized identity becomes a means of resisting injustice. This is what Mounk (2023) argues can lead inexorably to a trap in which an overemphasis on identity leads to rigidity and intolerance, rendering mutual influence unacceptable. In such a narrative, identity itself becomes how we express our citizenship and assert our human rights.

These competing and contested narratives are an integral part of the social and ideological context in which we ourselves are discovering, improvising, crafting and negotiating new and different practices and identities, in processes that are messy, incomplete, pragmatic and often emotionally and socially charged. That said, it is important to recognize that each narrative holds within it truths, which may resonate to some degree with the lived experiences and understandings that both coach and coachee bring into the coaching room.

### 2.2 Identity, identity work, and change

In this article, we conceptualize identity in terms of a socially constructed, reflexively created self (Foucault, 1988). This aligns with our interest in how the person forms, constructs, and attaches meaning reflexively to who they are, how they relate to others, and how that sense of self informs their decisions and actions. In coaching, reflexive questions about identity and the self invariably arise. Questions about how to perform as the leader one is expected or strives to be often take center stage for our coachees. We use the term “identity work” to refer to the processes of forming, maintaining, strengthening, repairing, or revising identity (Sveningsson and Alvesson, 2003; Watson, 2008). We concur with the view that identity itself is neither wholly ascribed nor entirely chosen, and that identity work is a dynamic and ongoing, process of negotiating and regulating the self, to produce a more or less coherent and distinctive identity.

A key driver of identity work seems to be in situations where we experience a threat to our current identity or sense of self. For writers such as Foucault, this is the constant and continuous working of becoming. Some identity researchers and scholars (e.g., Ibarra, 1999), emphasize the temporary transitions and changes that occasion threats to identity equilibrium. Breakwell (1986), suggests that when our identities are threatened, we use coping strategies to protect the integrity of these identities. He described strategies that people deploy at three different levels. Firstly, strategies of self-protection on the intrapsychic level. For example, when a coachee might seek to protect a sense of vulnerability in being a new entrant to the marketing profession, by banishing any inner doubts or self-critical thoughts about their professional prowess. Secondly, strategies that work on an interpersonal level, by changing relationships with others as a means of coping with threat. For example, the same coachee might refer to their qualifications and specialist knowledge in the domain of marketing as a way of asserting that they have a credible professional identity, in their dealings with people around them. And thirdly, intergroup strategies, encompassing different group levels and structures. These might include, for example, the coachee seeking out, or socializing with, other marketing professionals or visibly aligning themselves with marketing as a function or department within their organization.

We argue that relational coaching lends itself well to the exploration of SD and identity. We believe that working based on mutuality of relationship, our personal implication in the shadow side of our own psyches, and the shadow of the social order we live within, can engender multiple possibilities for change. A relational stance can help us to find ways to acknowledge, address, and work through the multi-dimensional impacts of oppression and SD in coaching. SD and dissonance may provoke threats to identity, but they can also offer the opportunity to explore the emotions and ideas that arise. Attending to what happens in the relationship between coach and coachee, and interrogating how both are implicated in oppression and privilege, can become valuable questions for exploration. Together, such explorations can also contribute to the re-working of identity, to what it means to be a leader and what the identity of a socially conscious or responsible coach, and indeed leader, implies for the actions we take and the choices we make.

## 3 Methods

Stimulated by the changing membership of our faculty team, the experience of lockdown following COVID, and the resurgence of the Black Lives Matter movement, we embarked, together with other faculty members of the Ashridge MSc in Executive Coaching on our own journey of critical reflection and exploration of identity, SD, and its impacts on our work. We have spent the last 2 years in monthly hour-long seminars and self-development work, culminating in a one-day team workshop. Together, these aimed to:

1. Offer peer support and supervision on a range of cases where we struggled to maintain a WA with some coachees in our executive-coaching practice and participants on our MSc program. Most of these featured race difference, though several featured gender or neurodivergence. We worked through a process of critical reflection, and review, analyzing the cases we brought for discussion and making joint decisions in some instances where distancing or escalation were a feature.
2. Provide an opportunity to undertake our own awareness-building, autoethnographic work and exploration of identity, SD, and its impact on our work. A foundational part of this work involved us drawing up implicated-subject statements.

We describe some of the key steps in our journey of critical reflection and exploration of identity, social difference, and its impact on us and our work as a faculty team below.

Our central problem statement posits that issues of SD can work against the underlying beneficence of coaching conversations and other developmental interventions. As our own learning journey as a faculty team had demonstrated, identity and SD can pose considerable challenges, especially when they are left unexplored. We sought to experiment with designing an approach that captured a responsive stance by the coach, of listening and responding moment-by-moment to the conscious and unconscious ways in which identity, SD, oppression and trauma present themselves and are configured in the coaching conversation and relationship.

We set ourselves the task of noticing the underpinning assumptions and narrative patterns in our work with our tutees and coachees, including the recurring debates about the importance of identity and lived experience of oppression and trauma on the one hand, and the denial of identity and SD as socially produced on the other. These debates give rise to polarized positions where the feeling of being constantly on the edge is palpable. We observed that our conversations with our coachees, tutees, and indeed with each other cycled between anger, distress, and silence. Resistance, reaction, and hesitation are all understandable mechanisms that keep us at a distance from seeing the operation of oppression and trauma inside ourselves, our social structures, and our relationships, making change and inclusion seemingly impossible at times. We also observed that under certain conditions an iterative process of conversation, exploration and “working through” the conflict and distress led to greater mutual understanding, empathy, and appreciation for the other. It seemed that the presence of SD and working through its meaning and impact, might itself be an indirect force for good. In some circumstances, making sense of the impact on relationships can bring people closer, and be a source of learning and compassion. Our own process of working through as a faculty team, seemed to follow this pattern. These divergent patterns resulting ultimately in a parting of the ways in some cases, and the successful maintenance of the relationship in others, seemed to reoccur in coaching and similar helping relationships.

As we have already mentioned above, we used implicated subject statements as a part of our explorations. These were statements which described significant experiences and cataloged the ways in which we felt ourselves to be implicated in historical and current social oppression. The implicated subject statement exercise (Kabasakalian-McKay and Mark, 2022) is based on the argument, made by Rothberg (2019), that the traditional binary categories of perpetrator and victim, do not sufficiently account for people’s complex involvement in historical and contemporary inequality. It follows from this argument, that regardless of our position, whether as oppressor, oppressed, or for that matter bystander or ally, we may find ourselves to a greater or lesser degree, unwittingly aligned with power and privilege, but not necessarily actively complicit in oppressive harm.

Sharing our experiences and perspectives with each other from the vantage point of being implicated in oppression, but also as oppressed, enabled us to explore our experiences of hurting and being hurt. Recognizing that these impacted our interpersonal dynamics, and our here-and-now conversations and experience, served as critical and profound moments of understanding. They helped us to gain a deeper appreciation of our own identities, and insight into how these are socially shaped and mediated.

Expressing our own complicated feelings honestly and bearing witness to those of our colleagues, helped us to bridge the distance and to heal ruptures in our own relationships. We felt closer and more connected as a result. The process also helped us to interrogate and embrace the disavowed elements of own behavior in our relationships- where social difference was involved. Placing this in a wider social context, which nonetheless invoked our responsibility- left us feeling more able to focus on the meaning, significance, and impact for the “other.” Rather than getting entangled in feeling hurt, guilty, or becoming defensive, we were able to acknowledge and subsequently let go of those less productive feelings, and to keep our attention and energy focused on the impact and experience of SD for the person concerned, and how this played out in various ways in their relationships.

The shared sense of connection led us to experience greater compassion for ourselves, for each other and for our course participants and our coachees. Ultimately, it led us to redefine our responsibility in these relationships. It was through our deep exploration of identity, SD and their distorting influences, and through the lens of the implicated subject, that we found ourselves able to attach new, less defensive meanings to our own identities. This “identity work,” this re-working of how we saw ourselves, suggested new and different perceptions of what our responsibility constituted, and new options for action that we could take. The process of working through the distance and disruption that the topic, and indeed the impact of SD on our relationships as a team, seemed to stem our impulse and tendency to retreat.

In the coaching relationship a retreat into difference, privilege and the woundings of history may make a true, open meeting of minds difficult. As coaches we are hindered in our work by our own identity retreat and the hidden operation of internal splitting and projection (Davids, 2011). These are processes which stand in the way of overcoming reductive identity polarization, the denial of social difference, and other sources of distancing in the coaching relationship.

In the sections below, we draw from our experience in approaching this problem head on. Some of our reporting is based on longer composite case studies, as well as shorter case vignettes. All details are completely deidentified and anonymized. For reasons of space, we have selected four composite case vignettes that represent the evolution of our learning, and which illustrate significant dimensions of our developing practice. We start with our work as a faculty team with Don, which we believe illustrates how SD and attempts to inquire and bring attention to the dynamics of gender difference can impact and resonate. The second and third case examples of one-to-one coaching, with Rahima and Milena respectively, illustrate how SD comes to be configured in the identities, we adopt, enact, and negotiate during the course of establishing and maintaining an executive coaching assignment. In the former, there was a retreat from identity and in the latter a mirroring and replay of SD in many ways. The fourth example shows the potential for working at depth, in the context of a strong WA, to recognize and honor the profound and inherently inescapable nature of othering and oppression.

We use our findings to develop a model of the impact of SD on the WA in coaching, and to propose some ways that coaches can be explicit about and explore SD, and address distancing, misunderstandings and disagreements that can arise as a result of SD.

## 4 Findings and analysis

### 4.1 Experiences with SD in our teaching and coaching practice

#### 4.1.1 Case Vignette I. Impact and dissonant resonance. Don

As a teaching team we all played a role in working with Don. He had initially expressed a degree of enjoyment and satisfaction in being part of a cohort of mature consulting professionals undertaking a postgraduate level diploma in coaching practice. He actively participated in the workshop learning and in practice and supervision groups. Don had trained as a clinical social worker in the United States, a training more akin to psychotherapy training in the U.K. and elsewhere. He had grown up in the deep south, a region with a history of conservativism and patriarchy. During the group-based teaching sessions Don came across as a curious and thoughtful practitioner who was generous in sharing his therapeutic experience with colleagues, and open to reflecting on his experience.

However, during a one-to-one review of his first personal assignment on the course, he voiced a strong objection to a question posed by his tutor, Carol, when she gently inquired about his experience of working with a woman coachee. He was very upset about being asked the question and repeatedly asserted that the coachee’s gender was completely irrelevant. Moreover, he refused to consider or discuss his view on the situation or his stance toward his coachee any further. Carol invited him to talk through his perspective, and to explore the session on working across difference that was an integral part of the taught program. Don said to her that Carol was being “sexist,” then refused to continue the conversation and ended their call. In the weeks that followed, Don did not respond to Carol’s messages, and he refused to speak to her or to Jessica, his group supervisor. Carol was left with a feeling of being silenced, shut down, and Jessica was frustrated that Don had unilaterally withdrawn from their supervisory relationship without explanation and without discussion. Don subsequently sought out Adam, another member of the teaching team and asked if he would take over as his tutor.

Adam offered to give Don some time to explore what had led him to ask for a change of tutor, and when they met, was struck by the sense of being ascribed a very particular role in Don’s set-piece-account of events. Adam felt that he was expected to support Don’s appeal to “male solidarity” and was curious that Don was reluctant to discuss the rupture with Carol or why he wanted to change tutor. Don was adamant that he truly did not see any point in exploring what had “gone before” and did not understand why he wasn’t free to choose a new tutor. Adam was left feeling perplexed and frustrated. Don felt angry and let down. For him it was a matter of good customer care that he should be able to work with the tutor of his choice, and that, as a fee-paying mature professional student, he was entitled to make a judgment about who was most competent and best suited to help him successfully complete his course work. Don went on to raise several complaints at management levels in the institution about the quality of teaching, supervision and tuition offered by Carol, Jessica and another woman member of the team. When these complaints were not upheld following a formal investigation, he asked that he be compensated by being granted an extension to complete his certification. Don felt he had come to us “more or less ready” to get his certificate. If anything, he felt he had been an ideal participant: psychologically informed and literate, and able to help other less experienced participants on the course. He also felt that he had acted responsibly to begin with, keeping his disappointment and misgivings about the program to himself and focusing on completing the coursework requirements.

#### 4.1.2 Case Vignette II. A retreat from identity. Rahima

One of us (a woman of color), briefly coached Rahima (another woman of color), who had worked as a senior director in a large media and advertising organization, where she had found herself increasingly marginalized and excluded from major decisions about content and the representation of people of color, all this in the midst of sincere talk of social inclusion and social justice. Rahima felt that the whole organization, though apparently well informed, was nonetheless riddled with protective practices and defensive maneuvers, which silenced her voice and left her out of key decisions. These elements had worked together to maintain the colonial order of things.

I started working with Rahima, after she had left the organization, having set up her own company, which had expanded fast, and was now growing further. Rahima had sought me out as a coach in part because of our similar social experience- both being women of color. During our chemistry conversation and during the first of the coaching sessions, we explored Rahima’s experiences of being silenced and stopped from producing more socially relevant creative content in her previous organization. We also talked about her current enterprise, where she had been able to find expression and had produced innovative and award-winning work. Rahima’s main goal for the coaching was to build trust with her senior leadership team. We had talked about the shared experience of marginalization, and initially Rahima expressed her delight and relief that there was no resistance or hesitation from me in seeing her experiences of being silenced through the lens of colonialism and racism. As our coaching progressed, the urgency of the need to be accepted and validated by the members of her racially mixed senior leadership team (SLT) and her white investors seemed to become paramount, and Rahima subsequently withdrew from our coaching contract.

During the latter part of our work, I was struck by how often Rahima used skin-related metaphors. For example, referring to trust with her SLT being only “skin deep,” to working with partners who did or did not have “skin in the game,” to herself as being “thick skinned.” This led us into an interesting exploration about identity and connection, and her lack of connection and trust with her own family of origin. Her history and heritage, as a Kenyan Asian, had been characterized by dislocation, multiple separations, and a profound sense, during childhood, of being misunderstood. In the coaching relationship with me, and in the work, Rahima felt torn between her ability to be truly herself, and her wish to belong, to get on and to ensure that her business survived. She decided against renewing our coaching contract and did not want to have a session to close our work together. Instead, I wrote her a working note as a way for me to reflect on the coaching sessions we had had, in which I shared my observation that we did not seem to be as aligned or in tune as we might have been. It was through a brief email exchange which followed, that Rahina acknowledged that she found it difficult to talk about herself, to locate herself and her identity, except through her assertive endeavors in service of underrepresented communities through her work.

#### 4.1.3 Case Vignette III. Mirroring and replay. Milena

One of us, a woman coach of color, worked with a highly successful executive and CEO of a food manufacturing company, Milena (a white woman). She was hardworking and pragmatic, proud of what she characterized as her fearless, “no nonsense” approach, and her ability to solve complicated problems. Milena came from a modest working-class family and had been academically, professionally, and socially successful. Educated at Cambridge, she had had an international career as a consultant with several prestigious consulting firms prior to moving into food manufacturing, where she was now in her third successive role as CEO of a brand within the same parent company. On first encounter, she came across as fiercely protective of her identity as a woman leader in what she described as a deeply patriarchal industry. She could not fully articulate why she had chosen me as her coach, except to say that she a vague inkling that working with someone who wasn’t “white and English” like her was probably “a healthy thing to do,” especially as she was being called upon to show more leadership in the “diversity, equity and inclusion space.” This, together with strengthening her people orientation, were two core aims we agreed for our coaching work. Forming an alliance with Milena felt to me to be hard going. Being asked questions about her experience and her aspirations frustrated her, as she felt these were irrelevant, detracting from working on her objectives. She was scathing in her evaluation of our first session, which she felt had been a waste of her time, reflecting that she felt there was such a gap between us, especially as I had never been a private sector CEO, and I was most certainly not “English like her.”

Milena was adamant that she wanted to continue working with me nonetheless. Our subsequent session was similarly tricky and felt quite ineffective. Milena ended by making a veiled quip about my competency as a coach. When I asked her directly about this, she simply said: “well it might be me.” Her comment struck me as sincere- signaling that she may have felt in some way not competent herself. Our third session proved to be something of an emotional roller-coaster, and a turning point. I confronted Milena about what I experienced as her need to see me as “not okay, not competent, and not White and English like her, and therefore not good enough in her books.” Her reaction was to apologize “unreservedly” for her remarks about Englishness, and later to express her sense of incomprehension about having to talk about herself in coaching, and to make way for “all these diversity and inclusion initiatives at work.” She could not help feeling cornered and manipulated, at her workplace with her boss, but also in coaching with me. The one paralleled the other. It was through exploring her feeling that she was in some way being maneuvered by me and through inquiring into what my SD represented for her, that we came to build a greater sense of connection and trust. She told me that she was frustrated by my manner: my slow delivery, and vague personal questions. What did I want her to say? Why did I ask her about her feelings? Why did I assume that she found uncertainty scary, when in fact she found it exciting, liberating? I acknowledged that my approach had unsettled her, and we agreed to frame and structure our sessions differently hereafter. Another concern for her, was that in her view, I had made a song and dance about my identity; something she had no interest in and could not care less about. Not only did she think my SD was irrelevant, but I was someone of no consequence in her career or her world. I shared my feelings of deep consternation and sadness at her lack of interest and curiosity in me as her coach, her lack of interest in relating with me. Her other angry tirades focused on the shortcomings of others: their lack of capability, decisiveness, efficiency. Schoolchildren without aspiration: “gen Z snowflakes” who demanded time off work to take their pet hamster to the vet, gay people who went on about their personal life in the office, Black people who played the race card, lazy leaders who let others “do the heavy lifting.” When I shared my impression that she seemed to see lots of deficits in others and asked how she saw her own capabilities and shortcomings, Milena broke down. Through her tears, she repeated, that she “just got on with it, that she always plowed through.” It transpired that her boss was, as she put it, “toxic.” Behind closed doors, he was demeaning, bullying, and demanding, making it clear that if her company (effectively a division of the larger corporation) did not make its ambitious cost-base reductions and sales targets by the end of the next quarter, her job would be at risk. Publicly, he was emollient and encouraging, projecting himself as a gender and diversity champion. Inwardly, she felt her position was utterly precarious, that all her achievements and hard-won socio-economic status among the elite and privileged might, after all, count for nothing. The more acutely she experienced a sense of alienation and inauthenticity in role, the less comfortable and authentic she felt as a leader, at the helm of a foods manufacturer.

A chance remark at the beginning of a session, when Milena was explaining why she had been delayed visiting a customer, seemed to pave the way into an interesting co-inquiry. The client, a large supermarket chain, had been experiencing rising rates of shoplifting and earlier that day, Milena had witnessed an older woman of color being apprehended by the store security guard. It was this incident which had made her late for our session. She was keen to process what she had seen, and to make sense of her subsequent conversations with the staff at the store. The managers and security team had told her about the growing problem of gangs commissioned to shoplift, mainly items of food, to order, but also the growing numbers of people, many of them apparently refugees or young Black people shoplifting in the supermarket. Referring to the latter groups, Milena could not understand why “they” would risk a criminal record or what their motives were. She supposed it was done for laughs, or a thrill. Nor could she understand why the gangs shoplifting to order were not more interested in relatively higher value items such as spirits, rather than food. Moreover, she was perplexed that the security guard had “gone after” that older woman- who, come to think of it, looked a bit like me, she added. I shared with her, that I was often followed around by store security guards, and that I have been stopped on occasion, and asked if I had any unpaid items on me. We explored the social context, the factors potentially driving the theft of food, cost of living among them, as well as the social perceptions and prejudices about those groups shoplifting or indeed possibly wrongly suspected of doing so.

Milena opened our next session, several weeks later with the words: “I’m sorry about that security guard thing that happens to you.” When I asked “why sorry?” Her reply was astonishing. “I’ve looked you up. You’ve been here almost all your life, you have studied, worked and practiced in this country forever. You’ve gone into some different companies in your time. It looks like you have done a lot of training too, to do this kind of work. Why do you get security guards following you around when you shop? Why should what you look like make you a suspect?” This was a truly affecting and transformative moment in our hitherto strained relationship. When I later asked Milena what had changed for her, she thought that it was something about what she had pieced together about my life in the U.K., and what she had inferred from our coaching work. She had come to the conclusion that we were not so different after all. I had stuck it out, and that’s what she had done. She had stuck out being bullied at Grammar school, at Cambridge, and climbing up the greasy pole in her first consulting company job.

In the sessions that followed Milena wanted to work on changing the pervasive metaphor and life script of “sticking it out,” she wanted to explore the possibility of more agency and choice in her career, shaping what kind of leader she wanted to be. We circled back to the topic of diversity, equity and inclusion on several occasions. Milena remained suspicious about “political correctness” and the potential for superficial leadership gestures and virtue signaling. At the same time, she felt personally compelled and professionally obliged to start some change initiatives. After attending a sustainable leadership program, we explored what Milena took from the experience and what it meant for our contract. What emerged in the identity work of crafting what kind of sustainable leader she wanted to be, were the twin notions of being energetic and pragmatic. This crystallized for Milena into potentially practical solutions to two business problems around labor shortage and food waste. She returned to the problem of shoplifting again, looking at it through the eyes of the sustainable leader that she increasingly saw herself as becoming, and the energetic and pragmatic leader that she had always been. This is how she summed it up in one of our later sessions: “It’s common sense. We’re crying out for people to work in our industry, and we are also deeply shameful when it comes to food waste. What’s more I do not think it’s right that all those people are being forced or feel they need to shop lift. To my knowledge, my grandparents did not steal food to survive, but they did go hungry so that my parents could eat.”

#### 4.1.4 Case Vignette IV. Honoring impact and implication. Pravati

One of us (a white man) had been coaching Parvati (a woman of color) for several months before she felt safe enough to share the story of her childhood and adolescence. She told her coach about how she had been born in a provincial town to first-generation immigrant parents. She lived in fear of her father, who drank heavily and had a violent temper. Her mother was fiercely ambitious for her and somehow found the money to pay for a private education. She did well academically, becoming close friends with the only other girl of color in her year group. They supported one another in coping with the casual racism and discrimination that was widespread in 1970s Britain.

Listening to Parvati’s story, I was reminded of my own experience of that time. A few years older than her, I remembered the impact of Enoch Powell’s “Rivers of Blood” speech of 1968, and how I would joke with school friends using the denigrating labels that were used on television and part of everyday language during that time (terms such as “Paki” and “nig-nog”). I felt confident enough in my relationship with Parvati to gently share this experience, I described it as a “Me too” moment, but from the other side. I told Parvati that I knew what she was talking about, because I was also there and part of the dynamic. I shared my feelings of sadness and shame for how I had played along, acknowledging that I had not know any better at the time. There was a calm, peaceful quality to the dialogue as we both noticed how our experiences were being held in our bodies. Both shed tears. As we did so, there was a deepening sense of trust in our relationship.

### 4.2 Reflecting on our learning from case experiences

#### 4.2.1 Don

As we explored our interactions with Don and examined our individual responses to the events that had unfolded, our explorations crystallized around three distinct themes. The first related to responsibility. Inevitably, we questioned our responsibility and contribution to the situation: what might we have missed? And what could we have done differently? Second, we felt a deep sense of frustration that Don had not been able to stay directly in conversation or to sustain a relationship with us. And third, we had found it very difficult to feel empathy with his actions and with his experience. What little he had shared, about growing up as a white man in the segregated southern U.S., suggested a legacy of social if not interpersonal entanglement with the traumas of racism and of gender segregation. Far from having a resilient WA with Don, we found ourselves in an unproductive stalemate. Our conception of good practice in talking about the potential significance of SD was apparently at odds with Don’s needs and expectations. We were at an impasse, where the WA was temporarily compromised and potentially seriously imperiled. Our respective perspectives were determined by our own experience and investment in particular expectations and outcomes.

#### 4.2.2 Rahima

In the case of the coaching with Rahima, the apparently similar experience of SD between coach and coachee, turned out to be “skin deep”; operating at a socio-cognitive level of understanding, rather than the deeper level of attunement and connection arguably required to forge a more solid WA. On the coach’s part, seeking to join with Rahima on the grounds of a common experience and a socially similar identity, appeared to be a misstep, possibly reflecting an overly coach-centric perspective, and an over-emphasis on apparent demographic similarity. This may have overlooked a world of difference in experience, and led to the coach missing the all-important impact, and contribution of a traumatic disappointment in Rahima’s formative familial experience. The coach’s early emphasis on similarity and an apparently shared experience of SD, seemed ultimately to invite an avoidance of alliance on Rahima’s part and to create distance between the coach and coachee. Adapting to earlier familial and possibly intergenerational trauma and wounding, had perhaps created a fragmentation and splitting in which the regulation of identity for Rahima rested on the stability of an external, agentic, “work” self. It seems likely that Rahima construed a threat to her identity as a professional creative leader and woman of color, which centered on a business rationale, together with the psychological need for her to belong. She came to author her sense of self as a preferred identity anchored in the occupational professional world rather than rooted in heritage and SD. In this scenario, the apparent resolution and such identity work that Rahima undertook, was located in her work enterprise and occupational identity and the work of belonging-as-socially-different within the dominant culture.

#### 4.2.3 Milena

In this scenario the coachee seemed to perceive and experience her coach as considerably socially different, and the earlier part of their work was fraught with misunderstanding. The prospect of connection and mutual empathy seemed remote. Instead, their interactions appeared to recapitulate the oppressive dynamics of “othering” and to parallel the dehumanizing impacts of leadership pressures and workplace bullying. In this case, Milena seemed to experience, at least initially, SD, and talk of SD almost as an assault on her personal and professional identity and status. Yet it was one of her self-described identity attributes, that of “sticking it out,” that helped to kindle her self-compassion, and her compassion-for-the-other, beginning with an empathy for her coach’s “life story,” in which she saw a “life-script signature” similar to her own. Her coach found that there was real value in staying with her client’s experience and in finding ways to tolerate the sense of emotional assault that was palpable in Milena’s antagonism toward her. Another important element that contributed to establishing a closer relationship, was the coach’s empathic response to Milena’s pain and consternation at feeling imperiled as a privileged white woman and as a leader. It was through the mutual recognition of elements of each other’s experience that coach and coachee were able to bridge the gap of perspective and lived experience they encountered.

Milena seemed to be faced with identity threat through insecurity and instability at work, to which she arguably responded by conforming, keeping the integrity of her preferred identity as an elite achiever and successful executive intact. However, this strategy did not seem to banish the threats to her identity, and Milena consequently modified her previously fixed story about herself. In this case, the coaching space and the coaching relationship itself operated as a containing, holding environment in which apparently polar identity positions, negative feelings, and positive appreciation could be borne. The disorientation and unease occasioned by several factors during the period of the coaching contract, provided three key opportunities for change. The first was the forging of a WA, through which Milena came to see her coach in a different light and to kindle positive feelings toward her as a person and a coach. For the coach’s part, she had to get beyond the coachee’s strong discriminatory negative projections onto her and a strong wish to withdraw from the assignment, and to feel compassion for her coachee’s experience. The second was the opportunity for Milena to rework her identity, softening her rigid narrative as all-successful, superior and elite, to one of a leader who was more in touch with a broader and richer perspective and her role. The third opportunity that opened up through the coaching, was Milena’s capacity to discern a role for her leadership in the shoplifting scenarios that were not strictly within the scope of her operations. In this scenario, it seems that the work of identity resulted in the leader considering a broader responsibility and the potential to incorporate social action as part of her role.

#### 4.2.4 Pravati

In the vignette describing the session with Pravati, the post-colonial history of immigration and racism came into the room, as experienced by coach and coachee on two different sides of the same story. Instead of seeing the coach as a supportive parent figure as she had done before, Parvati felt that she could relate to him as a human being, person-to-person. Her coach in turn experienced a kind of brother–sister bonding with Parvati. They both felt that they had established a more genuine connection, close to what Clarkson (2003) called the “real” or “I/Thou” relationship, albeit a professional one. They were both able to understand and articulate something about their story and acknowledge the powerful feelings of distress brought to the fore in the present. It was the coach who acknowledged the unhealthy forces of oppression and SD that had acted on their experience, and he was honest in sharing what they had impelled in him. Both were confronted in that session, with the deep impact of trauma which separated them in SD, and the asymmetrical impacts of oppression, but also connected them in the experience of the wounding at the same time. In this we see the profound respect the coach was able to hold for both sets of experiences and for the feelings engendered without side-stepping the implications of responsibility, power and hurt.

### 4.3 Reflecting on our coaching practice

Polarized positions, identity politics and racist thinking are based on a particular (paranoid schizoid; Klein, 1946) mindset, where we split and project in relationship (see, e.g., Davids, 2011). We create a dichotomy, between our own denigrated and prized attributes, and we unconsciously allocate the prized, positive attributes to ourselves and our “in group” and identify (project) the denigrated attributes with the other, the “out group.” In this process of “othering,” we construct the other as inferior and less human, less worthy than me/us. This is thought to provide the short-term pay-off of simplifying the world for us, enabling us to attach more easily to those who appear to share our identity. Additionally, because members of the same group generally find themselves placed relatively similarly in the same social field (i.e., they are exposed to similar information from the same perspective), their prototypical beliefs, assumptions, and stereotypes are more often than not, very similar, which reinforces those ideas. In the longer term, we pay a high price both socially and psychologically, in that we erect a screen between ourselves and others, and even between our own ideal self and our shadows- the undesirable and unwanted parts of ourselves. This inevitably limits our capacity to change, adapt and grow, given that our context is one of adults operating in a complex, social reality.

Workplace coaching is not, in any case, served by the dichotomies that come with a paranoid-schizoid mindset. Rather, it is a place for equanimity and integration; supported by critical reflection and reparative *cum* generative exploration. It is somewhere to build bridges of understanding and compassion between different parts of ourselves, between us and our stakeholders, and between the stories of those other players in the workplace—for example, colleagues and bosses—and the coachee’s own narrative. In coaching, we often find ourselves creating the conditions to imagine how we may be “implicated” in the narrative and in a multiplicity of positions within (or ways of looking at) the narrative.

Taking a relational stance as coaches arguably privileges understanding what happens between people in their human relationships. At one level, that means paying close attention to the here-and-now “micro” influences and manifestations of splitting and projection. At another, to the “macro” social influences and impacts that the external social realities bring into the coaching room. By observing and tracking the relationship-level interactions and dynamics at play in the coaching encounter, we can identify them, and reflect on their possible meaning with our coachees, understanding what they may signify. By looking at the wider social and societal level impacts and conditions reflected in the coaching encounter and the conversations between coach and coachee, we can foreground these for consideration too. Both micro and macro relational issues of social difference, oppression, and identity, can become discussable themes to reflect on and learn from in coaching. It is in this way that a relational stance can bring the experiences and external realities of SD, oppression, and identity into view, making what is already implicit and influential, explicit and capable of being investigated, examined, and better understood. This is how we often facilitate inquiry into SD, at multiple levels of understanding: personal, interpersonal, and social- while making space for diverging and intersectional experiences of SD.

Thinking first about the coach’s approach, we identified several elements that enable coaches to move beyond splitting and reductive polarization, and to enter into a fuller, more genuinely two-person relationship with their coachees. Taking a position of *equitable mutuality*, can be a helpful starting point for the coach. We define equitable mutuality as a climate of mutual OK-ness, in which the coach adopts a leveling, “I’m OK, you are OK” position (Harris and Harris, 2012), and is in touch with the real-life consequences and impacts of SD. In addition, there is the need to recognize ourselves in the other, and to participate in the experience of the other. If (either/or, paranoid-schizoid) experience reinforces oppression, then it is in empathy and compassion that we can feel for and stand in solidarity with our coachee. Importantly, it is in exercising unflinching, critical self-examination that we can find the oppressor within and feel responsibility for the unwanted and disowned aspect of ourselves and by the same token, the social world we occupy. This is what leads us to recognize that we are implicated in the very outcomes we oppose (Rothberg, 2019), and consequently, in the positions and circumstances we oppose too. As we have noted above, Rothberg maintains, that as implicated subjects, we play crucial, if indirect roles in systems of domination and histories of harm. Recognizing that we all play a part in the bigger systems that have created and continue to perpetuate SD and its individual, relational, and broader sequalae, is not to say that everyone holds the same responsibility. However, that does not mean that we cannot take responsibility together for inquiring into how issues of SD impact our lives and for creating new ways of understanding and meaning making.

Secondly, in responding to the subject matter or themes arising in coaching, we found ourselves typically using models to further mutual understanding, or to bridge a divide or a growing distance between people. One such model we use, is the well-known drama triangle (Karpman, 1968), which can serve to illuminate the construction of a dynamic whereby people take up an absolute position in relation to others. For example, rather than seeing herself as entirely powerless in the face of a new development in her business, we might invite the coachee to see the construction of a triangular “drama” in which there are the roles of Perpetrator (the new development), Victim (herself) and Rescuer (the coach). In this way, we can help her see how she can just as easily construct herself as Perpetrator or Rescuer, as well as Victim. In this way, we are able to help our coachees (and indeed ourselves) appreciate how we might be *implicated* (that is to say, how we may be part of or complicit) in initiatives we do not agree with. This paves the way for being able to own the part we play in the situation. In coaching, this can be essential for creating a more empathetic view of the other, and a different perspective on oneself, both of which contribute to creating new patterns of mutual self-awareness and new ways of relating. It is important to be clear here, that we are advocating the use of the drama triangle as a model to shed light on our psychological and social tendency to oversimplify, exaggerate and thereby disown the unwanted parts of ourselves or indeed the parts that we may play, unawares, in the events which take place around us. We are not suggesting that coaches ignore or underplay the need for coaching to take account of the operation of oppression and power, and the all-too real lived experiences and impacts of SD. Rather, we are proposing that we invite a perspective that facilitates a proportionate and realistic recognition of the actual power, vulnerability, and responsibility that different parties may hold in the situation. Appreciating how we might be implicated can contribute to a shift in perspective, a “re-calibration” of how we see ourselves, the actual part we do play, and the potential to play a different role in the situations we find ourselves in.

Thirdly, by taking account of the socially mediated nature of the self as an active creator of meaning and driver of change, we came to appreciate the pivotal importance of attending explicitly to the domains of identity and self in our coaching encounters. This involves careful exploration of the coachee’s experience and beliefs about their own and indeed others’ identity, as *an integral part of the coaching conversation*. It also requires that the coach commits to the same type of exploration as an integral part of their own self-development, whether in supervision or elsewhere. Additionally, it merits the coach’s close attention to the dynamics of identity as they evolve for the coachee. In other words, the identity work that the coachee does as they come to attach different self-meanings and behave differently in interactions with the coach and with others, crafting and enacting “credible” identities in respect of SD or in their role as leaders.

In summary, our findings provide support for our first hypothesis, that a positive WA plays a pivotal role in enabling coach and coachee to work through the challenges posed by SD. Our second hypothesis, that issues to do with SD can be better managed if they are explicitly surfaced, named and reviewed by coach and coachee, is partially supported by the findings. It seems that the explicit naming, surfacing, and reviewing of issues relating to SD in coaching is necessary, but there may be several additional factors that contribute. A closer analysis and review of our practice points to the need for the WA and coaching relationship to be a positive one, or to have the potential to grow. The existence or potential for a positive coaching relationship seems to provide the pre-conditions which enable SD to become an integral theme, a “natural” part of the coaching conversation, and a source of learning and growth.

In addition, our analysis suggests a number of principles that may additionally inform and support how coaches can establish and optimize a dialogue about SD in coaching. These include:

1. Holding the coaching space as integrative and generative, to bridge and build understanding, rather than reinforce unexamined or given perspectives. This enables coaching to encompass multiple perspectives, positions, and complex social realities.
2. Taking a relational stance to coaching. This involves examining experiences of the here-and-now of the coaching relationship, as well as the wider external social conditions, and the interconnections between the two. It also means inquiring openly about SD, and co-creating a shared understanding of SD, oppression, and identity.
3. Cultivating an attitude of equitable mutuality. This facilitates a climate of OK-ness and leveling, and for empathy and compassion to develop.
4. Using models and interventions that encourage perspective-taking. These bring potential connections between self and others, and the possibility of implication, into view.
5. Taking account of the self as socially constructed and mediated, capable of stimulating change and regulating identity, through identity work.

### 4.4 A model for working with SD

Coaching can undoubtedly result in powerful and effective change, though it is not clear precisely what processes are involved in the course of the journey. It has been modeled as a more or less predictable journey from initial fears, hesitations and discomfort, to love, satisfaction and effectiveness (De Haan, 2022), whereby the coaching contract delivers on pre-agreed outcomes. In our modeling of an implicated, relational stance toward SD we assume that “staying in coaching” will be beneficial, because, as a minimum, it sustains an opportunity for continued dialogue, for the kindling of compassion, and the regulation of identity. Our case experience suggests that awareness and understanding of the meaning and potential significance of SD, that is, our implicated selves, may be particularly important at the start of the coaching relationship. In addition, inquiry with empathy can co-create understanding, and mutual self-awareness about the impact of SD on the relationship dynamics between coach and coachee. These are activities which can contribute to the WA. The process of understanding distancing and rupture, and working through the experience, that is, recognizing and owning our individual part in it, can lead to further understanding of identity and SD- leading to greater mutual empathy. This deeper understanding and empathy can further strengthen WA and the coaching relationship. We believe that in many cases, relational safety and the potential for creative discovery are likely to increase, as coach and coachee stay the course, making progress toward achieving the agreed coaching objectives more likely. This means that staying in coaching is crucial, and for this we need WA.

**Figure 1:**
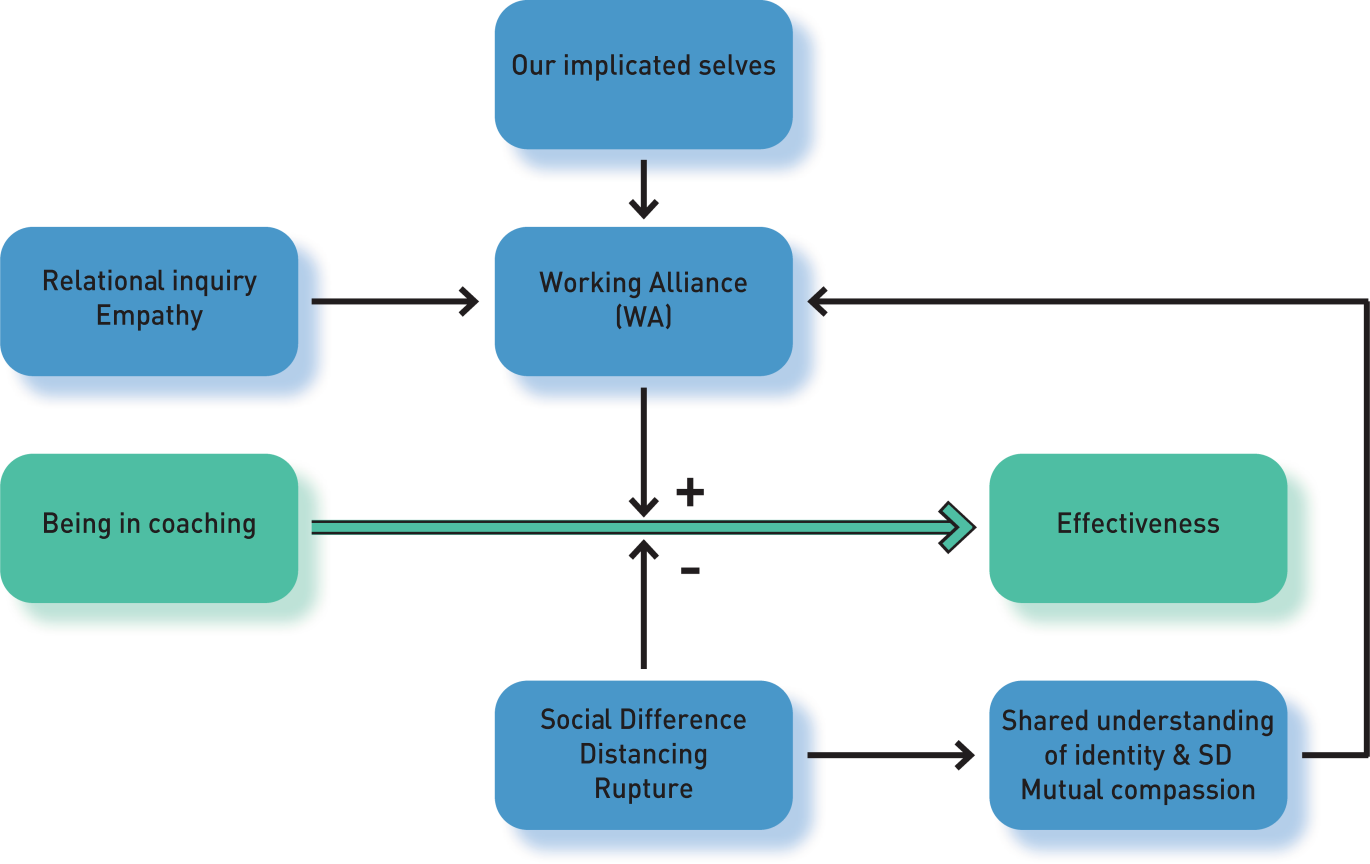
A model for working with SD and WA in coaching.

An illustration of how WA can counter the potential for SD to lead to distancing and rupture in the coaching relationship and facilitate the continuation of coaching, thereby enabling the coaching contract to achieve the desired outcomes, can be found in Figure 1. It is important to point out, that although the figure is comprised of static “boxes,” each of the boxes is there to represent a dynamic (whether of a process or a relationship) that will evolve over time but remain in the indicated relationship to the other dynamics. Therefore, it is important to bear in mind that this is a highly simplified and abstracted model.

### 4.5 Implications for practice

There are several factors which have been shown to predict a positive WA in coaching, including positive transference, motivation self-efficacy, and social support. However, there are also many factors that can render the WA unsafe, out of kilter, and *a priori* negative, particularly in the early stages of the coaching relationship. We have found that when it comes to identity and SD, the issues may require a stronger bond of trust and attachment between coach and coachee, than it might for other types of workplace or executive coaching topics. Given the potential for SD and identity to be inherently challenging in the coaching encounter, we would recommend that the coach actively invest in building WA, and other aspects of the coaching relationship by:

1. Maintaining the baseline conditions of being reliable and predictable.
2. Being open and motivated to discuss SD, oppression, and identity.
3. Setting an expectation of inquiry and discussion about SD and identity as an integral and “ordinary” part of coaching.
4. Inquiring about, tuning into, empathizing, and emotionally connecting with the coachee’s experience.
5. Working at whatever level of alliance proves possible, if the coach’s emotionally connecting behaviors are not reciprocated by the coachee.
6. Cultivating mutual trust, empathy, and support, and foregrounding unconditional appreciation to surface issues of SD and oppression, while implicating ourselves as coaches in these issues.
7. Co-creating a shared understanding of SD, oppression, and identity, while acknowledging ourselves as implicated, expressing our own genuine emotions and responses openly.

## 5 Discussion

Identity threats and disturbances to established narratives about the self may occasion or reinforce psychological defenses and a paranoid-schizoid mind set, in which an integrated, regulated view of the self and others becomes difficult to achieve. The consequent dichotomies and binary positions militate against the formation and the maintenance of an affirming, Adult-to-Adult relationship and a positive WA between coaches and their coachees. Our model suggests that investing in building the WA and strengthening the relationship between coach and coachee is paramount. It is the WA, and the strength and depth of bonding and trust in relationship, which seems to make it possible to work through the challenges of distancing and rupture SD can present. If coach and coachee are able to sustain their alliance and relationship, and stay in coaching, then they can not only acknowledge and address the issues of SD, but they can also co-create a shared understanding of self, and their implicated selves, along the journey to delivering the primary objectives of the coaching. This can often lead to identity work, whereby coach and coachee re-work their conceptions of themselves and their behavior. It can also contribute to a broader perspective on the self in relation to others, a deeper consideration of the meaning and impact of identity, SD, and inequity, as well as an ethically mature outlook and the potential to increase the scope of leadership responsibilities, in service of actions that may be more congruent with a wider social purpose.

Our article offers some ways to approach SD as part of coaching practice. These emphasize a relational stance, underscoring the importance of highlighting the interactions and influences of SD, on the relationship between coach-coachee. Talking about SD as it manifests and is reflected in the person and identity of both coach and coachee, and in the relationship between them inevitably presents a number of risks and challenges. That the benefits of doing so seem to be easier to realize in the context of a stronger coaching relationship, suggests that coaching practitioners may need to sustain a focus on building relational safety (working *on* the coach-coachee relationship), as well as a focus on bringing the here-and-now relational climate to bear (working *in* the coach-coachee relationship).

Furthermore, we suggest that making the influences of SD on, and in the coach-coachee relationship intelligible and available can be a valuable source of mutual understanding within the coaching relationship. We argue that this often serves to strengthen the WA and the coaching relationship, through mutual compassion and development of the capacity to stand with the experience of the other. We also emphasize the value of making the dynamic interplay between the self and the social world visible and amenable to change by using interventions which encourage perspective-taking and by exploring the coachee’s ideas about and implication in SD, identity, and oppression, including their own. The exploration of this dimension of self-in-interplay-with-the-social-world offers another way in which coachees (and coaches) can view and learn about themselves, expanding the coaching space to offer opportunities for identity work and the development of self.

This exploratory paper has drawn on a few representative cases to examine the impact of SD on WA in coaching, and to suggest that SD is better managed explicitly and as part of the process of coaching itself. Our broad conclusion is that coaches need a positive WA and relationship with their coachees if they are to work through the challenges presented by SD in coaching, and that this can be a worthwhile and productive endeavor. By working through, we are referring to the psychological processes of awareness, recognition, understanding and change that can take place within a helping relationship such as coaching. Our experience suggests that a resilient WA and coaching relationship mediates many important processes involved. We would suggest invoking the notion of “enough-ness,” to borrow Winnicott’s concept of the “good-enough” parent (Winnicott, 1958). Although ruptures, impasses and other difficulties may impact the quality and strength of the WA and the coaching relationship, it is nevertheless capable of being established, rekindled, and repaired. In other words, the WA, and the coaching relationship prove to be resilient enough to be a holding environment to contain the attendant feelings, ideas associated with exploring SD and identity. Moreover, the process of exploring SD and identity may itself further strengthen WA and the coach-coachee relationship.

## 6 Conclusion

Our article proposes that in attending to the myriad ways that SD manifests and impacts in the process and content of coaching, particularly in the here-and-now of the relational coaching encounter, the WA and other aspects of the coaching relationship are key. We also suggest that coaches may be most effective when they can establish a climate of OK-ness and show deep regard for the experience and impact of oppression, and the ways in which they are implicated. Psychologically, and socially we may disown, discount, and oversimplify SD. In doing so we may take away the opportunity to understand and face into it, forfeiting the possibility of acknowledging its impact, and the potential for change. We propose drawing on Rothberg’s thinking (Rothberg, 2019), that we are inevitably implicated in issues of oppression and social differentiation, and implicated by extension, in the need to acknowledge and address them as an integral part of our coaching practice. A relational coaching stance involves intense and relentless tracking, paying active and persistent attention to the here-and-now of the coaching conversation to make SD explicit, discussable, and potentially a stimulus for change. In this way coaches can inquire into the domains of the self, taking account of the coachee’s evolving identity work, and facilitate perspective-taking in service of a more generative and socially conscious, implicated practice.

As coaches, we need to find ways to negotiate identity and the experience and the impacts of SD, for ourselves and for our coachees. Far from being themes that sit outside the realm of coaching, whether spoken or not, they remain implicit and important, and they often limit or derail the progress and potential of the coaching endeavor. Our paper, which to our knowledge is the first of its kind, has examined the ways in which coaches and coachees can cultivate compassion and sustain a generative alliance and conversation in the face and aftermath of distance and rupture. It is not in retreat from the relational unease which SD and social differentiation may occasion in coaching, in the familiarity of an entrenched position or narrative of identity, nor is it in despairing at the wider context of social injustice, denialism and ideological confusions about the ethics of equity but rather, in confronting these tensions head on, and in approaching, inquiring into, and explicitly engaging with the attendant dynamics and difficulties that we discover a freedom to coach at a deeper level.

### 6.1 Limitations and future research

Our study has several limitations. For example, although we have grounded our inquiries in existing theoretical and research insights, we have gleaned further theoretical and practical insights from a small, albeit deliberate and distinctive sampling of our work. We selected a small number of cases to illustrate aspects of our evolving practice as coaching educators and practitioners, and how we fell into patterns which we had begun to identify through our processes of reflective peer-group supervision. Future studies could helpfully investigate our findings and proposed model across a larger number of cases, in different settings, possibly utilizing multimethod approaches to combine observational, interview and case study data. Triangulating across data sources would help to reveal more about the interplay between and relative influence of different factors such as matched social identity of coach and coachee; timing of explicit review of SD; and the particular approaches taken to repairing rupture and overcoming impasses in the context of SD and distancing.

There is considerable scope for future research to build on our exploratory study, to further explore, extend and refine our findings in several possible directions. Executive coaching holds considerable promise as it continues to evolve as a dialogic and relational practice and extends the theoretical and knowledge base it draws on. We believe there are some particularly important areas which would merit further inquiry and research. One concerns the presence, influence and expression of heightened emotions such as guilt and shame. The open expression and sharing of strong emotions by coachee and coach is one that warrants investigation. Another important area relates to the apparently mediating role played by the WA and other aspects of the coaching relationship, as both a pre-condition for a constructive exploration of identity and SD and also a consequence of successfully working through of difficulties in alliance arising from the impact of SD in coaching. The relationship between WA, other relationship factors and SD can be empirically tested. Additionally, relationship factors separate from WA, such as trust and mutual empathy would warrant further investigation. Studies involving close discursive analysis of coaching interactions concerned with SD might shed light on the micro-conversational and micro-relational turns and exchanges that contribute to deepening connection and trust.

## Data Availability

The data analyzed in this study is subject to the following licenses/restrictions: the raw case material data which were used to inform our analysis and findings are held by the members of the Ashridge Centre for Coaching Faculty Team in line with our institutional data collection and privacy requirements, and professional coaching standards and codes of conduct. Each case record contains identifying details, together with sessional notes relating to the individuals concerned, but it is only accessible to the individual executive coach who worked with the individual, and in the case of course participant data, to the course directors. All records are stored securely, and held in strict confidence, in line with requirements of the Data Protection Act 2018 and the GDPR as retained in UK Law by the Data Protection, Privacy and Electronic Communications (EU Exit) Regulations (2019) for personal and sensitive data. Protection and anonymization of subject identity, the data presented in this article has been carefully anonymized to protect the identity of the individuals concerned. All the case examples are drawn from past cases, and none relate to any active or ongoing engagement with a coachee or course participant. Where we have presented unfolding episodes and events, in the longer narratives, we have done so by creating a composite picture, in which several cases have been combined into a single case study. All names, incidental information, and reference to occupations, locations or organizations been changed or, where they might assist identification, have been omitted altogether. In the case of the shorter vignettes we have used, these have been completely condensed and anonymized. The authors have applied the Human Givens Institute HGI “can you look them in the face test?” by ensuring, that for each example the answer to the question: “Would we be able to show the case study to my course participant or coachee, in the knowledge that the account is fair and accurate and that their identity is adequately protected?” was a definitive, consistent and consensual “yes!” (https://www.hgi.org.uk/about-hgi/ethics-and-conduct/hgi-ethics-conduct-policy/guidelines-writing-and-use-case-histories). The only exception to complete deidentification relates the Ashridge Coaching Centre Faculty Team and the account of their peer supervision and continuing development sessions. However, all names have been changed and the description of events does not attribute any individual or detail any specific episode. Moreover, they are fully aware about the information that we have shared in the article. With regard to consent in the case of course participants and coachees, we considered that obtaining retrospective informed consent for publication, was both impracticable and potentially unethical, as to do so would involve the “imposition” of a post-hoc relationship, beyond the boundaries of the original, contracted relationship. Restrictions the raw case material data from which we derived the composite and condensed case examples used in the article are restricted for reasons of subject confidentiality and privacy and cannot be divulged or made available.
